# Nutrient Enemata

**Published:** 1907-02-16

**Authors:** 


### POINTS IN TREATMENT.

### NUTRIENT ENEMATA.

Rectal feeding must necessarily mean partial starvation of the patient, for it is not possible for the rectum and colon to absorb enough foodstuffs in 24 hours to supply the minimum number of calories required by the body in that time. Nevertheless there are circumstances in which it is impossible to feed by the mouth, and rectal feeding is resorted to as being better than no feeding at all. It has even been found possible to maintain life by nutrient enemata over periods of six weeks or more at a stretch. When rectal feeding becomes necessary, it is most important to give such enemata as will be most easily absorbed, whilst at the same time affording as large as possible a supply of calories of energy to the patient.

A healthy man, at rest in bed, requires something like 3,000 calories per diem to maintain his strength and weight; a sick man, in whom metabolism as a whole is below par, will probably not lose strength if the food he absorbs can yield 2,000 calories per diem; a woman may do with even less; perhaps as little as 1,500 calories per diem may suffice to prevent loss of weight and strength, though they would not allow of any gain in weight except under exceptional circumstances.

If 1,000 to 1,500 calories be the least amount of energy that the absorbed food must yield in order to maintain a patient even at a low ebb, the question arises: To what extent can this be supplied per rectum? It is clear that *nutrient suppositories* are practically useless. The total amount of energy they contain is almost negligible, and to give them as a food is to disturb the patient without feeding him at all.

A very usual nutrient enema consists of the yolk of one egg, a little salt, and peptonised milk to four or five ounces. Let us see how much energy this enema contains:—

| | | | Calories. |
|---|---|---|---|
| Yolk of 1 egg | Proteid, 38 grains or 2·5 grams | = | 10.0 |
| | Fat, ʒi. or 4.5 grams ... ... | = | 40.5 |
| Peptonised milk 4 ozs. | Proteid, ʒss. or 2 grams ... | = | 8.0 |
| | Fat, 45 grains or 3 grams ... | = | 27.0 |
| | Carbohydrate, 75 grs. or 5 grams | = | 20.0 |
| Salt 2 grams | | | |
| | | | 105.5 |

It would be necessary to give between 14 and 15 of these a day to supply 1,500 calories, even supposing the whole of each was absorbed. It is seldom possible to give more than six a day—*i.e.* one every four hours, so that the energy supply will not reach 650 calories.

This being so, it will at once occur to one that the amount in each enema should be increased. The question of how best to increase it is the difficulty. There are many obstacles in the way. In the first place, the rectum will not retain more than a certain volume of fluid at a time; and in the next, absorption of other things than salt and water is so slow that the first enema will not have been absorbed before the next is due, if the bulk exceeds a certain maximum. The bigger the enema the less often can it be given. If, however, large enemata at longer intervals will do as well as smaller ones more frequently, other things being equal, it is right to use the large injections; for the less often the patient has to be disturbed the better. It has been found that 9 oz. enemata can easily be given three times a day, and it is now the general custom to use them in preference to the 4 to 5 oz. enemata formerly given four-hourly.

This, however, does not supply the patient with more food energy than before. The 9 oz. enemata will not be absorbed if they are given more frequently than three times a day. Is it not possible to alter the ingredients of the enema, so that each may yield a larger amount of energy?

It may be asked, why not give more fat, which bulk for bulk yields more than twice as much energy as do either proteid or carbohydrate? Unfortunately the rectum, even under the most favourable circumstances, cannot absorb more than about ½ oz. of fat in 24 hours—*i.e.* about the quantity contained in the yolks of three eggs. It is therefore useless to put more than one egg yolk into each 9 oz. enema.

In regard to proteid, the peptonised foods of all sorts are well assimilated, but their prolonged use usually leads to irritation of the mucous membrane, and consequent non-retention of the enemata after a time. Gelatine, undigested milk, and cooked meats are hardly absorbed at all. Luckily raw meat juice and egg albumen are both absorbed comparatively easily and without being peptonised, and it is upon egg albumen and upon peptonised milk that we rely most for the supply of proteid in rectal feeding. Their rate of absorption, however, is slow, so that it is seldom possible to give more than 50 grams of proteid per diem in this way. For example:—

| | | Total. |
|---|---|---|
| Whites of 3 eggs | Proteid, 11 grams | 16 grams or ℥ss three times a day. |
| Peptonised milk 9 ozs. | " 5 grams | |
| | Fat, 3 grams or 45 grains. | |
| | Carbohydrate, 11 grams or 165 grains. | |
| Salt 3 grams or 45 grains. | | |

It is upon carbohydrates that one must chiefly rely in increasing the calorie energy of the food supply in rectal feeding. Starch is the best form in which to give it. Cane sugar is not good because it is absorbed unchanged. Dextrose can only be absorbed in dilute solution, because it has a high osmotic pressure. Starch, on the other hand, is colloid, and has no osmotic pressure; it is well absorbed from the rectum, whether given boiled or unboiled, and it is assimilated. It is easier to give it unboiled, because it does not then render the enema too thick for injection purposes. It can be added to any other form of enema that may be chosen, and the constituents of the enemata can be varied from time to time in a given case according to circumstances, but the relative heat values of any given enema forms the best criterion of their relative usefulness, provided each is retained and absorbed. The following are the comparative heat values of two 9 oz. enemata:—

*Egg and Milk Enema.*

| | | Calories. |
|---|---|---|
| Whites of 3 eggs | Proteid, 11 grams or 165 grains | =44.0 |
| Yolk of 1 egg | Proteid, 2.5 grams or 38 grains | =10.0 |
| | Fat, 4.5 grams or 68 grains ... | =40.5 |
| Peptonised milk 9 ozs. | Proteid, 5 grams or 75 grains | =20.0 |
| | Fat, 3 grams or 45 grains ... | =27.0 |
| | Carbohydrate, 11 grams or 165 grains ... ... ... | =44.0 |
| Salt 45 grains | | |
| | | 185.5 |

*Egg, Milk, and Starch Enema.*

| | | Calories. |
|---|---|---|
| Pure starch | Carbohydrate, 60 grams or nearly 2 ozs. ... ... | =240.0 |
| Whites of 3 eggs | Proteid, 11 grams or 165 grs. | = 44.0 |
| Yolk of 1 egg | Proteid, 2.5 grams or 38 grs. | = 10.0 |
| | Fat, 4.5 grams or 68 grains... | = 40.5 |
| Peptonised milk 4½ ozs. | Proteid, 2.5 grams or 38 grs. | = 10.0 |
| | Fat, 1.5 grams or 23 grains... | = 13.5 |
| | Carbohydrate, 6.8 grams or 100 grains ... ... ... | = 26.0 |
| Salt 45 grains | | |
| Water to 9 ozs. | | 384.0 |

The egg, peptonised milk, and starch enema is the better; and if it needs modification in a given case, the part to leave out is the peptonised milk, replacing it with water. Upon three such enemata as the above, the patient will receive 1,152 calories of energy per diem in the food; upon this the loss of weight and strength will be very much slower than it is upon enemata of egg and milk without starch.

In giving the enema, the patient must be lying upon the back, and it is very important that the buttocks should be raised to prevent the fluid from trickling down towards the sphincter ani and causing a desire to defæcate. To raise the buttocks, a pillow laid across the bed beneath them may suffice; or it may be necessary to raise the foot of the bed by blocks. No syringe is required; it is best to use a rather soft rubber œsophageal tube and a funnel. The tube should be filled with the fluid to be injected, at a temperature slightly above that of the body; when all air has been expelled, the tube is clamped, and the lubricated end inserted slowly as high up in the rectum as possible. The clamp is then released, and the fluid is allowed to run in at its own pace, assisted by raising the funnel slightly to a height of about three feet above the anus. It may take a quarter of an hour before the whole of the enema has run in. It is very important that no air should be allowed to get in. When the tube is removed the patient should remain quietly in the hip-raised position for some time afterwards. The slower the injection is given the more certain is it to be retained.

Two other points in connection with rectal feeding are highly important. The first of these is that the patient will receive far too little water if none is given in addition to the three 9 oz. enemata. More water must be given. Comparatively large enemata of normal saline—*i.e.* ℨiss. of common salt to 1 pint of water—can be retained as a rule if given slowly at body temperature; twice a day, separate from the nutrient enemata, 1 pint of normal saline at 100° F. should be administered per rectum. The second point is that the mucous membrane must be kept healthy and clean, and all unabsorbed residues, together with the natural excretion of the bowel, which continues even during starvation, must be removed by means of a large soap and water enema once a day. This evacuant enema may be given with the patient supine, as in the case of the others, but with this difference, that it should be given more quickly and with a syringe; a pint and a half to two pints may be required.

Six times during the 24 hours, therefore, will the patient have to be disturbed. The hours at which it will be most convenient to do this will vary with the conditions of the patient, the duration of sleep, and so on. It is seldom justifiable to wake a patient on any account. As near as may be, however, it is convenient to start the day by cleansing the bowel with a soap and water enema at 6 A.M.; a 9 oz. nutrient enema of starch, egg, and peptonised milk may be given at 8 A.M.; a pint of normal saline at noon; a second 9 oz. nutrient enema at 2 P.M.; another pint of normal saline about 6 P.M.; and the third nutrient enema between 8 and 10 P.M. Allowing for non-absorption of part of each enema, the patient will probably assimilate food enough to yield 1,000 calories per diem, that is, enough to prevent more than gradual loss of weight, when feeding by the mouth is temporarily impossible.

